# Extraordinary Molecular Evolution in the PRDM9 Fertility Gene

**DOI:** 10.1371/journal.pone.0008505

**Published:** 2009-12-30

**Authors:** James H. Thomas, Ryan O. Emerson, Jay Shendure

**Affiliations:** Department of Genome Sciences, University of Washington, Seattle, Washington, United States of America; University of Texas Arlington, United States of America

## Abstract

Recent work indicates that allelic incompatibility in the mouse PRDM9 (Meisetz) gene can cause hybrid male sterility, contributing to genetic isolation and potentially speciation. The only phenotype of mouse PRDM9 knockouts is a meiosis I block that causes sterility in both sexes. The PRDM9 gene encodes a protein with histone H3(K4) trimethyltransferase activity, a KRAB domain, and a DNA-binding domain consisting of multiple tandem C2H2 zinc finger (ZF) domains. We have analyzed human coding polymorphism and interspecies evolutionary changes in the PRDM9 gene. The ZF domains of PRDM9 are evolving very rapidly, with compelling evidence of positive selection in primates. Positively selected amino acids are predominantly those known to make nucleotide specific contacts in C2H2 zinc fingers. These results suggest that PRDM9 is subject to recurrent selection to change DNA-binding specificity. The human PRDM9 protein is highly polymorphic in its ZF domains and nearly all polymorphisms affect the same nucleotide contact residues that are subject to positive selection. ZF domain nucleotide sequences are strongly homogenized within species, indicating that interfinger recombination contributes to their evolution. PRDM9 has previously been assumed to be a transcription factor required to induce meiosis specific genes, a role that is inconsistent with its molecular evolution. We suggest instead that PRDM9 is involved in some aspect of centromere segregation conflict and that rapidly evolving centromeric DNA drives changes in PRDM9 DNA-binding domains.

## Introduction

Allelic incompatibility at the mouse PRDM9 (Meisetz) locus can cause hybrid male sterility due to failure in spermatogenesis [1]. Rare dominant nonsynonymous mutations in human PRDM9 may also cause failure in spermatogenesis (azoospermia, [2]), suggesting similar allelic incompatibility in humans. These data support a role for PRDM9 in an early stage of pre-zygotic hybrid incompatibility, consistent with a role in speciation [1], [3]. Targeted PRDM9 knockout in the mouse causes sterility in both sexes due to a block in meiosis I in both the male and female germ line [4]. The germ line arrest morphology resulting from PRDM9 knockout and from incompatible PRDM9 alleles are identical, suggesting that the incompatible alleles abrogate PRDM9 function [1], [4].

The PRDM9 gene in human and mouse encodes a protein with KRAB [5] and SET domains followed by multiple tandem C2H2 zinc finger (ZF) domains near the C-terminus [6], [7]. The PRDM9 SET domain region confers histone H3(K4) trimethyltransferase activity, consistent with activity as a transcriptional activator [6]. The function of the KRAB domain of PRDM9 has not specifically been studied, but in other ZF transcription factors it is known to recruit histone deacetylases and histone H3(K9) methyltransferase, suggesting activity as a transcriptional repressor [8], [9]. Since the ZF domains are the only DNA-binding domains in PRDM9, it is likely that they confer the DNA-binding specificity of PRDM9. Because of its histone modifying activity and DNA-binding domains, it has been assumed that PRDM9 encodes a transcription factor that regulates other genes important for germ line meiosis but there is no direct evidence for such a role.

The structure of ZF domains of the type found in PRDM9 bound to DNA is well-established [10], [11], [12]. Tandem ZF domains confer DNA-binding specificity in a modular manner, with sequential ZF domains binding sequential 3 nt DNA sequences in target sites. Each core ZF domain is typically 21 residues long and consists of a conserved framework of amino acids that coordinates and positions a highly variable nucleotide contact region. Adjacent core ZF domains are joined by a conserved 7 amino acid linker region, which makes a DNA phosphate contact and coordinates adjacent zinc fingers. Within the core ZF domain, nucleotide contacts are made by an eight amino acid turn-helix that occupies the major groove of DNA. Three amino acids make the major nucleotide contacts and adjacent residues may contribute additional contacts and influence the positioning of the major nucleotide contacts. The positions of all these amino acids are highly conserved and can be directly inferred from ZF domain sequence.

We report analysis of molecular evolution of the PRDM9 gene based on sequence comparisons between primate species and on human polymorphisms.

## Results

### Primate Divergence and Positive Selection

**Figure 1:**
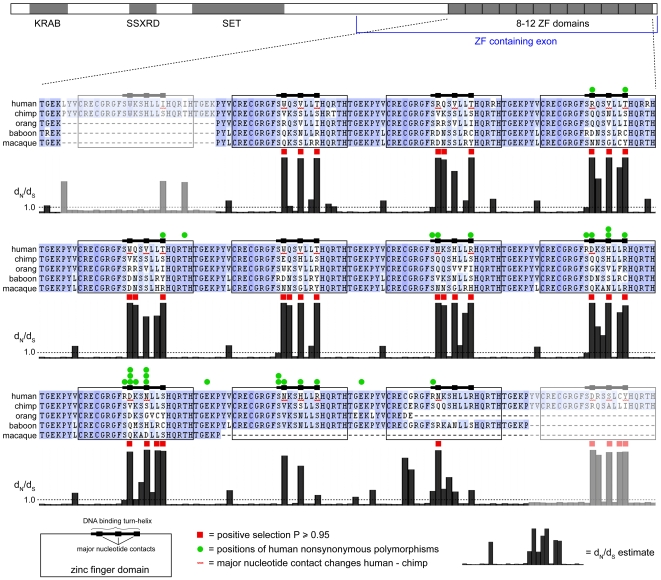
Sites of positive selection in primate PRDM9 genes. Schematic of PRDM9 protein showing KRAB, SSXRD (SSX repression domain), SET, and ZF domains. The ZF domain region of five aligned primate proteins is shown expanded and split into three sections, with blue shading proportional to amino acid conservation. The 12 human zinc fingers are boxed with thickened lines showing the DNA-binding turn-helix and its three major nucleotide contact residues. Below the alignment is a histogram of the Bayes-Empirical-Bayes estimate of d_N_/d_S_ ratio for each codon as computed by codeml. Filled red squares above the histogram indicate codons where the P-value for positive selection was 0.95 or more. Filled green circles indicate the positions of all known nonsynonymous human polymorphisms; positions with more than one mark indicate more than one distinct nucleotide change. Wavy red lines between the chimp and human indicate positions at which the reference protein sequence for the two species differ. Regions of the alignment with only two sequences are paled to indicate that the d_N_/d_S_ estimate is poorly informed.

We identified PRDM9 orthologs in primates and analyzed their molecular evolution. Most of the PRDM9 gene is well conserved in sequence, but the ZF domains are highly divergent. For example, in the 12 ZF domains of human and chimpanzee PRDM9 proteins, 28 of the 36 major nucleotide contact residues differ (Figure 1), despite a genome-wide average nucleotide divergence of 1.2% and protein divergence of 0.12% [13]. To study selection acting on primate PRDM9 genes, we performed maximum-likelihood analysis of synonymous and nonsynonymous codons in the human, chimpanzee, orangutan, macaque, and baboon. Most of the 894 codons in the PRDM9 alignment are characterized by negative (purifying) selection, indicated by a low estimated d_N_/d_S_ value (frequency of nonsynonymous change relative to synonymous change). However 32 codons had a high estimated d_N_/d_S_ value that reached statistical significance for positive selection (P>0.95). Of these 32 codons, 26 encode major nucleotide contact residues in ZF domains and all 6 other codons are immediately adjacent to major nucleotide contact residues. Several additional codons in DNA-binding turn-helix regions also had high estimated d_N_/d_S_ values that did not reach statistical significance. Divergence in the DNA-binding turn-helix regions is obvious by simple inspection of the protein multiple alignment (Figure 1). In addition to rapid divergence in the DNA-binding residues, there are several changes in the number of ZF domains among the five primates. All of these changes are the result of precise insertion or deletion of entire ZF domains, consistent with generation by unequal cross-over events. These changes are also expected to affect DNA-binding specificity. A high degree of amino acid divergence in the DNA-binding turn-helix region and changes in ZF domain number were also observed in other mammals, including rodents (data not shown).

The strong evidence for positive selection in nearly all of the DNA-binding domains and the fact that human and chimp PRDM9 differ so sharply in their major nucleotide contact residues suggests directional selection on PRDM9 to rapidly change DNA-binding specificity. Maximum-likelihood analysis also suggests that similar selection is acting on every branch of the primate tree (data not shown). The remainder of the PRDM9 sequence is conserved throughout mammals and even in more basal lineages including non-mammalian chordates and echinoderms [7], indicating that a highly conserved function is tethered to a rapidly evolving DNA-binding specificity.

### Human Polymorphism

**Figure 2:**
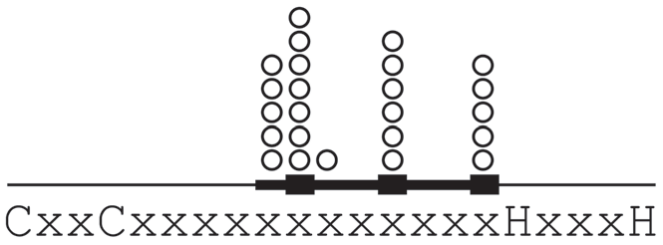
Human nonsynonymous polymorphisms according to position in their zinc finger. The amino acid sequence below shows the defining C2H2 zinc coordinating residues and their invariant spacing. The thickened line shows the DNA-binding turn-helix and its three major nucleotide contact residues. The open circles indicate the positions of each distinct nonsynonymous polymorphism, which are strongly associated with DNA-binding residues.

We assessed single-nucleotide polymorphisms (SNPs) in the coding region of human PRDM9 reported in dbSNP130, from 16 individual exome sequences generated by next-generation sequencing ([14] and this study), and from a large study of PRDM9 coding SNPs in Japanese men [2]. Of 35 distinct SNPs, 31 are nonsynonymous, indicating an exceptionally high population diversity in PRDM9 protein sequence. The positions of the 31 nonsynonymous SNPs are remarkable (Figure 1 and Figure 2): 28 of 31 affect ZF domains or the linkers between ZF domains, and 24 of these 28 affect residues in the DNA-binding turn-helix of ZF domains. In the entire PRDM9 protein of 894 amino acids, only 96 amino acids are in a DNA-binding turn-helix region (8 in each of 12 ZF domains), indicating a highly significant enrichment of nonsynonymous SNPs in turn-helix residues (24/31 vs. 96/894, P<0.0001 by Fisher's exact test). Taken together with the strong positive selection acting at the same class of sites, this pattern suggests an ongoing series of partial selective sweeps affecting PRDM9. In the study of Japanese men, allele frequencies were determined for 21 SNPs. Two of these 21 were found only once in patients with azoospermia; neither affects a ZF turn-helix. All of the remaining 19 alleles were common in the Japanese population (>1% frequency) and all 19 affect a ZF turn helix. Of the 12 nonsynonymous SNPs found in non-Japanese populations, only one is identical to a Japanese SNP. This lack of overlap suggests that both the Japanese and non-Japanese PRDM9 polymorphisms arose very recently.

### Zinc Finger Homogenization

**Figure 3:**
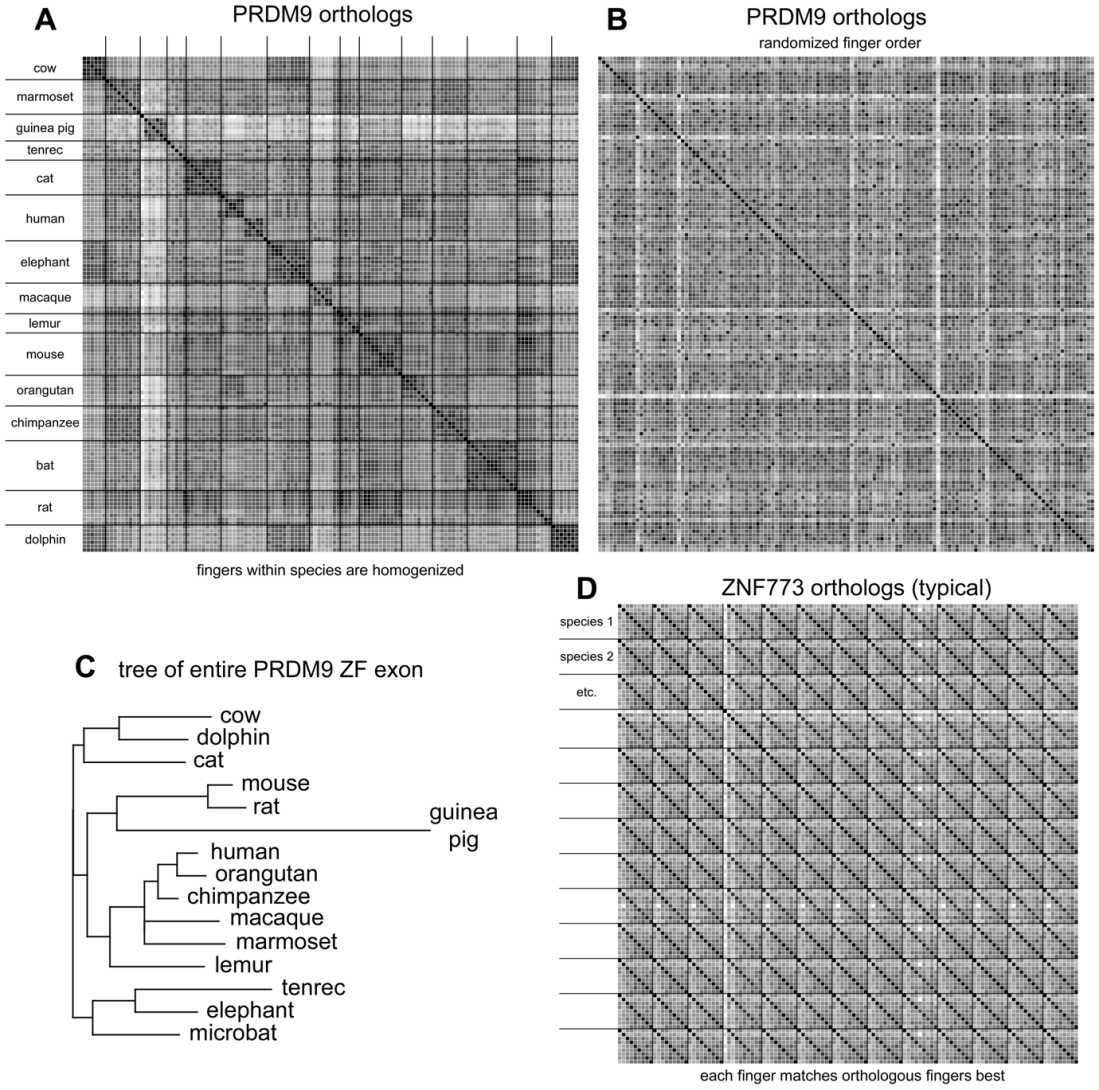
Zinc finger sequences are homogenized within genes and divergent between genes. Panel A. Self dot plot of all tandem 21 amino acid zinc fingers from PRDM9 orthologs in all 15 species with 5 or more zinc fingers. Dot shading corresponds to sequence similarity using the BLOSUM62 matrix, scaled so that the lowest scoring match is white and the highest scoring is black. Lines separate fingers from each species. Sequences are arranged by their taxonomic name and within each gene fingers are in order by position (N-terminal zinc finger first). Panel B. The same PRDM9 fingers with their order randomized, showing that the appearance of self-similarity in panel A is significant. Multiple randomizations were inspected; this one is typical. Panel C. A maximum-likelihood tree of the PRDM orthologs based on the protein encoded by their entire ZF encoding exon. The protein tree approximates the species phylogenetic tree, supporting orthology of the PRDM9 genes. Panel D. Self dot plot of all 21 amino acid zinc fingers from 13 ZNF773 orthologs (representative of other KRAB zinc finger genes, data not shown). All species except one have 9 zinc fingers (the additional divergent zinc finger in armadillo (species 4) is N-terminal and not tandem with the 9 orthologous fingers). Each zinc finger is closely related to its orthologous fingers from the other species and divergent from the other zinc fingers in the same gene. The species in figure order are cow (species 1), dog (species 2), marmoset (etc.), armadillo, horse, cat, human, macaque, rabbit, baboon, orangutan, chimpanzee, and ground squirrel. Because many of the PRDM9 genes come from low-coverage assemblies it is difficult to find a representative zinc finger gene from all of the same species as shown in panel A. The species are domestic cow (*Bos taurus*), common marmoset (*Callithrix jacchus*), domestic dog (*Canis familiaris*), guinea pig (*Cavia porcellus*), armadillo (*Dasypus novemcinctus*), lesser tenrec (*Echinops telfairi*), domestic horse (*Equus cabalus*), domestic cat (*Felis catus*), human (*Homo sapiens*), African elephant (*Loxodonta africana*), Rhesus macaque (*Macaca mulatta*), mouse lemur (*Otolemur garnettii*), domestic mouse (*Mus musculus*), domestic rabbit (*Oryctolagus cuniculus*), Hamadryas baboon (*Papio hamadryas*), Sumatran orangutan (*Pongo pygmaeus abelii*), chimpanzee (*Pan troglodytes*), flying fox bat (*Pteropus vampyrus*), Norway rat (*Rattus norvegicus*), thirteen-lined ground squirrel (*Spermophilus tridecemlineatus*), and bottlenose dolphin (*Tursiops truncatus*).

**Figure 4:**
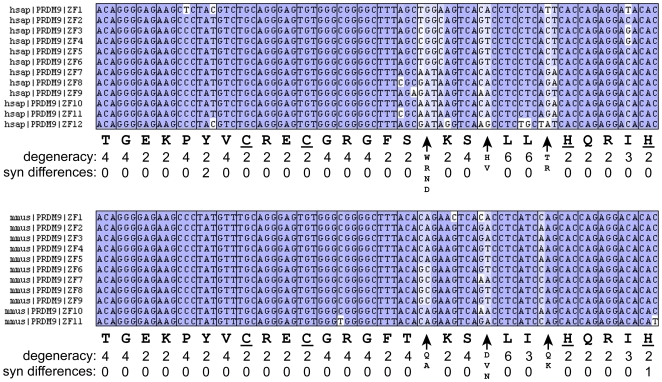
Lack of synonymous site differences among fingers. Codon alignment of the 12 human (top) and 11 mouse (bottom) zinc fingers including the upstream 7-codon linker region (reference genome sequences). Blue shading is proportional to the frequency of the residue in its aligned column. For all codons, except the three major nucleotide contacts, the predominant encoded amino acid is shown below the codon, with the zinc-coordinating residues underlined. The next two lines indicate the number of possible codons for the predominant amino acid (degeneracy) and the number of synonymous changes observed among any of the fingers. The striking lack of synonymous divergence between fingers strongly supports recombination among fingers. At the major nucleotide contact residues (arrows) all amino acids encoded by at least two fingers are listed.

We noticed that zinc fingers within the PRDM9 gene in each primate tended to be similar in sequence, despite the rapid divergence among species. We investigated the generality of this pattern by identifying putative PRDM9 sequences from 19 sequenced mammalian genomes (including the primates in Figure 1). The 19 sequences all encode multiple ZF domains with the same spacing and arrangement as that in human and mouse. The number of ZF domains ranges from 3 to 20 with an average of 8.3. A dot plot of ZF amino acid sequence similarity within genes is shown in Figure 3. With the possible exception of tenrec, intraspecies ZF domains are clearly more similar than between species. These results are consistent with interfinger recombination resulting in homogenization of most of the ZF sequence. Homogenization among ZF domains in human and mouse is even more striking at the level of DNA sequence (Figure 4). For example, of the 28 codons that make up a single ZF domain plus linker, 19 encode the same amino acid in all 12 human ZF domains. All 19 of these codons are 2-fold or 4-fold degenerate, but only 2 of 228 possible synonymous differences are found among the fingers. Similarly, in the mouse only 1 of 264 possible synonymous differences is found. All 3 of these synonymous differences occur in the first or last zinc finger, consistent with partial recombinational isolation of terminal ZF domain sequences.

It is likely that such recombination events contribute to human population polymorphism as documented in Figures S1 and S2. Briefly, 28 of 31 distinct human SNPs found in ZF domains could have arisen by recombination with other ZF domains. Given the general homogeneity of sequence in the aligned ZF domains (Figure 4), this correlation is clearly significant. Rapid divergence in PRDM9 genes could be facilitated by such recombination events, which can allow spread of new advantageous mutations to other fingers [15]. We speculate that it is this process of spreading new advantageous changes by recombination that drives the homogenization of other sequences in PRDM9 ZF domains.

## Discussion

In summary, PRDM9 sequences across mammals show rapid divergence specific to the DNA-binding turn-helix region of their ZF domains and there is an exceptionally high level of nonsynonymous human polymorphism in the same classes of sites. What could account for these patterns? All other domains in PRDM9 are conserved among species, suggesting that a conserved biochemical function is tethered to a rapidly evolving DNA-binding specificity. The expected histone modification activities of PRDM9 (histone deacetylation and histone H3(K4) trimethylation) suggest a role in transcriptional regulation. However transcription factors are generally characterized by highly conserved DNA-binding domains, yet these are the regions where PRDM9 is most rapidly evolving. In contrast to transcriptional regulation, centromere structure and function is associated both with regulated histone modification states [16], [17] and with rapidly evolving DNA sequences [18], [19], [20]. A favored model for the rapid evolution of centromere sequence is the centromere-drive hypothesis, in which selfish centromeres compete to segregate to the oocyte during female meiosis [19], [21]. Centromere drive is potentially deleterious to the host by causing skewed sex ratios or male sterility, effects that may be balanced by observed rapid evolution in genes encoding centromere associated proteins [22].

The internal structure and boundaries of functional centromeres are strongly associated with histone modifications and with centromere-specific classes of histones [16], [17]. PRDM9 has the potential to regulate two of the known centromeric chromatin-associated histone modifications. First, human and *Drosophila* centromeric chromatin is hypoacetylated on histones H3 and H4 [17]. PRDM9 encodes a KRAB domain, which is well-established to recruit histone deacetylases via the KAP1 protein [23]. Though the KRAB domain of PRDM9 has not itself been studied, it is very similar to other KRAB domains and is probably the evolutionary origin of the huge family of KRAB zinc finger transcription factors [7]. Second, histone H3(K4) dimethylation is associated with centromeric chromatin, whereas H3(K4) trimethylation is often found at the borders just outside of centromeric chromatin [17]. The SET domain of PRDM9 has been shown to have H3(K4) trimethyltransferase activity (converting dimethyl H3(K4) to trimethyl H3(K4)). Thus PRDM9 could function to limit the extent of core centromeric chromatin by helping to define the borders of di- and tri-methyl histone H3.

Centromere drive is expected to be limited to the germ line, with the most obvious site of action being meiosis in the female. The only phenotype of PRDM9 knockout in the mouse is arrest at prophase of meiosis I in both sexes, consistent with various specific roles including recombination and chromatin condensation in preparation for metaphase. Our hypothesis for PRDM9 function clearly predicts that the PRDM9 protein will be physically associated with the centromere during meiosis I and that it will function there to moderate centromere drive via histone modification.

## Materials and Methods

### d_N_/d_S_ Analysis

Complete or nearly complete PRDM9 coding sequence was obtained either from available gene predictions (human, chimpanzee, orangutan, macaque) or from a genewise [24] prediction based on the human PRDM9 protein (baboon). Codons were aligned guided by a clustalw [25] protein alignment (default parameters). The codeml program from the PAML suite [26] was run on the codon alignment without gap removal using model 7 and model 8 (three starting omega values with unconstrained added omega class, plus one run with the added omega class constrained to 1.0). Evidence for positive selection was overwhelming (e.g. an 85.2 difference in log-likelihood for model 8 with unconstrained omega relative to model 8 with omega constrained to 1.0). The Bayes-Empirical-Bayes d_N_/d_S_ estimates came from the codeml “rst” output file and P-values came from the standard codeml output (both with unconstrained model 8).

### SNP Identification

Human SNPs were ascertained in three ways. First, a publication provides extensive information on human coding-sequence alleles and frequencies in a Japanese population [2]. Second, dbSNP130 was queried to obtain all known PRDM9 coding SNPs. Third, novel coding SNPs were ascertained by deep sequencing of 12 human exomes [14] plus an additional 4 exomes sequenced by the same method. Table S1 summarizes information on all the SNPs, including genome position, nucleotide change, and source. The haplotype configurations of the SNPs are unknown. Haplotter summary statistics in the PRDM9 region show no obvious signs of recent population-specific positive selection [27].

### Ortholog Identification

We wanted to obtain the PRDM9 ZF coding exon from available mammalian genome assemblies. Divergence in PRDM9 ZF sequences combined with large C2H2 zinc finger gene families throughout mammals made PRDM9 identification based on these sequences impossible (data not shown). For low coverage assemblies, the unique upstream coding exons were likewise useless because they were often not present on the same contig as the ZF exon. However, there is a conserved unique protein sequence upstream of the ZF domains in the ZF-containing exon (see Figure 1) that appears to be diagnostic for PRDM9 genes in mammals. We used this protein sequence as a tblastn [28] query of all available mammalian genome assemblies. The DNA corresponding to the best tblastn matches from each genome were extracted along with 1 KB of DNA downstream of the match. These DNA sequences were translated and tested for encoding multiple ZF domains in-frame and downstream of the diagnostic sequence. Finally, candidate sequences that passed this test were translated and a maximum-likelihood tree was made to confirm probable orthology of the sequences (see Figure 3). As expected because of incomplete assemblies, a PRDM9 ortholog was not found in all species, especially those with 2-fold coverage. In most cases additional confirmation that the sequence is a bona fide PRDM9 gene was obtained as follows: the marmoset, macaque, baboon, orangutan, and chimpanzee genes are complete or nearly complete in their assemblies and are clearly syntenic to human PRDM9; the rat gene is complete and syntenic with the mouse PRDM9 gene; the cow gene is nearly complete; and the cat, mouse lemur (*Microcebus murinus*), dolphin (*Tursiops truncatus*), and bat (*Pteropus vampyrus*) genes have an upstream exon on the same contig that matches the next upstream human exon from PRDM9. Finally, all the sequences except tenrec show clear homogenization of their ZF domain sequences, a very unusual character among tandem ZF domain genes (data not shown). The tenrec sequence is included based on the protein tree, but it should be regarded as a questionable ortholog assignment because it shows no other shared PRDM9 characters. Higher primates have a partial duplicate of the PRDM9 gene (PRDM7) but it completely lacks the tandem ZF domains of PRDM9 and is thus easily distinguished.

## Supporting Information

Figure S1 Generation of 6 SNPs by recombination. Alignment of the overlapping regions 18489–18657 and 18405–18573 of PRDM9 (numbering is genomic position relative to the ATG start codon). Colored dots indicate nucleotide differences between the two stretches. Gene conversion of PRDM9 18489–18657 (top line) with sequence from 18405–18573 (bottom line) could result in changes in one or any combination of the highlighted residues. In fact, each of these six changes appears as a SNP in the Japanese population [2], as shown by the inset. Allowing for these six nucleotide mismatches, the total region of identity available to support recombination extends 228 nt (an extra 54 nt 5' and 6 nt 3' of the sequence shown).
(0.73 MB TIF)

Figure S2 Generation of other SNPs by recombination. Alignment of all 12 ZF domains from human PRDM9, with arrows indicating the possible source (arrow tail) and target (arrow head) sites for generation of 20 SNPs by recombination. The arrow head is numbered to indicate the allele correspondence as given in Table S1 (gene position). In each case the target nucleotide in the SNP allele matches the source nucleotide in the reference allele. Numbers without superscripts correspond to Japanese SNPs [2]; numbers with an “rs” or “js” superscript correspond to hapMap130 alleles or alleles reported here respectively. Of the 31 distinct SNPs (28 nonsynonymous) that affect ZF domain sequences, the variant nucleotide of 28 is found at the homologous position of one or more other fingers, indicating that they could arise by recombination (rs72477009-17995, rs6875787-18013, Irie-18097/rs71578786-18097/js1-18097, Irie-18109, rs56256550-18245, rs58979818-18246, rs56001636-18266/js2-18266, Irie-18327, Irie-18329, Irie-18330, rs58945509-18339, rs55862350-18340, Irie-18350, Irie-18413, Irie-18414, Irie-18415, Irie-18416, Irie-18417, Irie-18423, js4-18423, Irie-18424, rs61051796-18495, Irie-18497, Irie-18498, Irie-18507, Irie-18518, Irie-18579, Irie-18635). Eight of these SNPs are not diagrammed on the figure for the sake of visual clarity. The remaining 3 SNPs cannot arise by recombination with any other finger from the reference sequence (Irie-17918, js3-18548, js5-18542).
(5.61 MB TIF)

Table S1 List of human polymorphisms in PRDM9. Summary of positions, nucleotide changes, data source, and other information for human SNPs analyzed. NOTE - single large table on multiple PDF pages.
(0.14 MB PDF)

## References

[1] Mihola O, Trachtulec Z, Vlcek C, Schimenti JC, Forejt J (2009). A mouse speciation gene encodes a meiotic histone H3 methyltransferase.. Science.

[2] Irie S, Tsujimura A, Miyagawa Y, Ueda T, Matsuoka Y (2009). Single-nucleotide polymorphisms of the PRDM9 (MEISETZ) gene in patients with nonobstructive azoospermia.. J Androl.

[3] Coyne JA, Orr HA (1998). The evolutionary genetics of speciation.. Philos Trans R Soc Lond B Biol Sci.

[4] Hayashi K, Yoshida K, Matsui Y (2005). A histone H3 methyltransferase controls epigenetic events required for meiotic prophase.. Nature.

[5] Bellefroid EJ, Poncelet DA, Lecocq PJ, Revelant O, Martial JA (1991). The evolutionarily conserved Kruppel-associated box domain defines a subfamily of eukaryotic multifingered proteins.. Proc Natl Acad Sci U S A.

[6] Hayashi K, Matsui Y (2006). Meisetz, a novel histone tri-methyltransferase, regulates meiosis-specific epigenesis.. Cell Cycle.

[7] Birtle Z, Ponting CP (2006). Meisetz and the birth of the KRAB motif.. Bioinformatics.

[8] Margolin JF, Friedman JR, Meyer WK, Vissing H, Thiesen HJ (1994). Kruppel-associated boxes are potent transcriptional repression domains.. Proc Natl Acad Sci U S A.

[9] Pengue G, Calabro V, Bartoli PC, Pagliuca A, Lania L (1994). Repression of transcriptional activity at a distance by the evolutionarily conserved KRAB domain present in a subfamily of zinc finger proteins.. Nucleic Acids Res.

[10] Pavletich NP, Pabo CO (1991). Zinc finger-DNA recognition: crystal structure of a Zif268-DNA complex at 2.1 A.. Science.

[11] Elrod-Erickson M, Rould MA, Nekludova L, Pabo CO (1996). Zif268 protein-DNA complex refined at 1.6 A: a model system for understanding zinc finger-DNA interactions.. Structure.

[12] Kim CA, Berg JM (1996). A 2.2 A resolution crystal structure of a designed zinc finger protein bound to DNA.. Nat Struct Biol.

[13] The Chimpanzee Sequencing Analysis Consortium (2005). Initial sequence of the chimpanzee genome and comparison with the human genome.. Nature.

[14] Ng SB, Turner EH, Robertson PD, Flygare SD, Bigham AW (2009). Targeted capture and massively parallel sequencing of 12 human exomes.. Nature.

[15] Thomas JH (2006). Concerted evolution of two novel protein families in caenorhabditis species.. Genetics.

[16] Allshire RC, Karpen GH (2008). Epigenetic regulation of centromeric chromatin: old dogs, new tricks?. Nat Rev Genet.

[17] Sullivan BA, Karpen GH (2004). Centromeric chromatin exhibits a histone modification pattern that is distinct from both euchromatin and heterochromatin.. Nat Struct Mol Biol.

[18] Malik HS, Bayes JJ (2006). Genetic conflicts during meiosis and the evolutionary origins of centromere complexity.. Biochem Soc Trans.

[19] Malik HS, Henikoff S (2002). Conflict begets complexity: the evolution of centromeres.. Curr Opin Genet Dev.

[20] Cellamare A, Catacchio CR, Alkan C, Giannuzzi G, Antonacci F (2009). New insights into centromere organization and evolution from the white-cheeked gibbon and marmoset.. Mol Biol Evol.

[21] Malik HS (2009). The centromere-drive hypothesis: a simple basis for centromere complexity.. Prog Mol Subcell Biol.

[22] Malik HS, Henikoff S (2001). Adaptive evolution of Cid, a centromere-specific histone in Drosophila.. Genetics.

[23] Friedman JR, Fredericks WJ, Jensen DE, Speicher DW, Huang XP (1996). KAP-1, a novel corepressor for the highly conserved KRAB repression domain.. Genes Dev.

[24] Birney E, Clamp M, Durbin R (2004). GeneWise and Genomewise.. Genome Res.

[25] Thompson JD, Higgins DG, Gibson TJ (1994). CLUSTAL W: improving the sensitivity of progressive multiple sequence alignment through sequence weighting, position-specific gap penalties and weight matrix choice.. Nucleic Acids Res.

[26] Yang Z (1997). PAML: a program package for phylogenetic analysis by maximum likelihood.. Comput Appl Biosci.

[27] Voight BF, Kudaravalli S, Wen X, Pritchard JK (2006). A map of recent positive selection in the human genome.. PLoS Biol.

[28] Altschul SF, Madden TL, Schaffer AA, Zhang J, Zhang Z (1997). Gapped BLAST and PSI-BLAST: a new generation of protein database search programs.. Nucleic Acids Res.

