# Supplementary figures and images for: Extraordinary Molecular Evolution in the PRDM9 Fertility Gene
Source: PLoS One. 2009 Dec 30;4(12):e8505. doi: 10.1371/journal.pone.0008505 (PMC2794550; doi:10.1371/journal.pone.0008505)

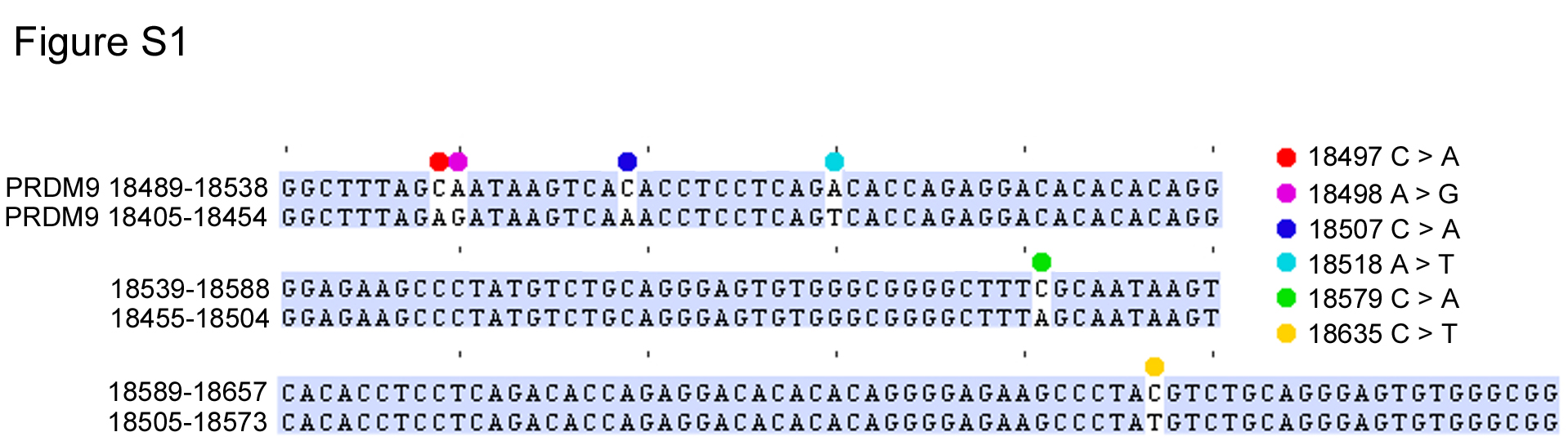

Supplement: Figure S1 — Generation of 6 SNPs by recombination. Alignment of the overlapping regions 18489–18657 and 18405–18573 of PRDM9 (numbering is genomic position relative to the ATG start codon). Colored dots indicate nucleotide differences between the two stretches. Gene conversion of PRDM9 18489–18657 (top line) with sequence from 18405–18573 (bottom line) could result in changes in one or any combination of the highlighted residues. In fact, each of these six changes appears as a SNP in the Japanese population [2], as shown by the inset. Allowing for these six nucleotide mismatches, the total region of identity available to support recombination extends 228 nt (an extra 54 nt 5' and 6 nt 3' of the sequence shown). (0.73 MB TIF) [file pone.0008505.s001.tif]

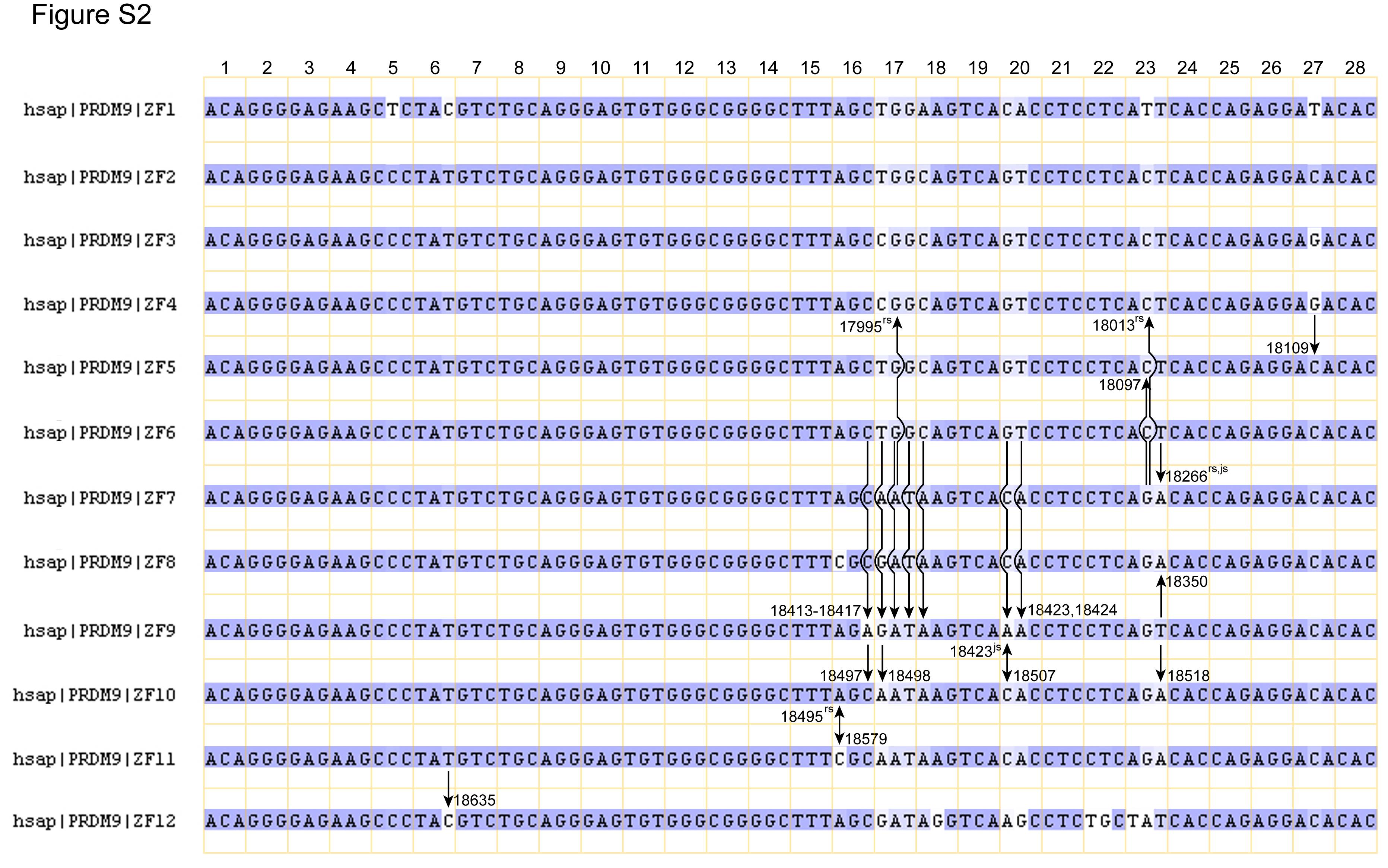

Supplement: Figure S2 — Generation of other SNPs by recombination. Alignment of all 12 ZF domains from human PRDM9, with arrows indicating the possible source (arrow tail) and target (arrow head) sites for generation of 20 SNPs by recombination. The arrow head is numbered to indicate the allele correspondence as given in Table S1 (gene position). In each case the target nucleotide in the SNP allele matches the source nucleotide in the reference allele. Numbers without superscripts correspond to Japanese SNPs [2]; numbers with an “rs” or “js” superscript correspond to hapMap130 alleles or alleles reported here respectively. Of the 31 distinct SNPs (28 nonsynonymous) that affect ZF domain sequences, the variant nucleotide of 28 is found at the homologous position of one or more other fingers, indicating that they could arise by recombination (rs72477009-17995, rs6875787-18013, Irie-18097/rs71578786-18097/js1-18097, Irie-18109, rs56256550-18245, rs58979818-18246, rs56001636-18266/js2-18266, Irie-18327, Irie-18329, Irie-18330, rs58945509-18339, rs55862350-18340, Irie-18350, Irie-18413, Irie-18414, Irie-18415, Irie-18416, Irie-18417, Irie-18423, js4-18423, Irie-18424, rs61051796-18495, Irie-18497, Irie-18498, Irie-18507, Irie-18518, Irie-18579, Irie-18635). Eight of these SNPs are not diagrammed on the figure for the sake of visual clarity. The remaining 3 SNPs cannot arise by recombination with any other finger from the reference sequence (Irie-17918, js3-18548, js5-18542). (5.61 MB TIF) [file pone.0008505.s002.tif]
