# Supplementary material for: Extraordinary Molecular Evolution in the PRDM9 Fertility Gene
Source: PLoS One. 2009 Dec 30;4(12):e8505. doi: 10.1371/journal.pone.0008505 (PMC2794550; doi:10.1371/journal.pone.0008505)
Supplement: Table S1 — List of human polymorphisms in PRDM9. Summary of positions, nucleotide changes, data source, and other information for human SNPs analyzed. NOTE - single large table on multiple PDF pages. (0.14 MB PDF) [file pone.0008505.s003.pdf]

name	genome position (hg18)	gene position	coding exon	ZF residue type changed	ref nt
rs7714612	23545357	457	2	NA	C
rs62342743	23553783	8883	4	NA	C
rs10042274	23556970	12070	5	NA	C
Irie-17353	23562253	17353	10	NA	G
Irie-17918	23562818	17918	10	NA	A
rs72477009	23562895	17995	10	MNC	G
rs6875787	23562913	18013	10	MNC	C
Irie-18097	23562997	18097	10	MNC	C
rs71578786	23562997	18097	10	MNC	C
js1	23562997	18097	10	MNC	C
Irie-18109	23563009	18109	10	zinc coordination	C
rs56256550	23563145	18245	10	MNCA	C
rs58979818	23563146	18246	10	MNC	A
rs56001636	23563166	18266	10	MNC	A
js2	23563166	18266	10	MNC	A
Irie-18327	23563227	18327	10	MNCA	C
Irie-18329	23563229	18329	10	NA	C
Irie-18330	23563230	18330	10	MNC	G
rs58945509	23563239	18339	10	MNC	C
rs55862350	23563240	18340	10	MNC	A
Irie-18350	23563250	18350	10	MNC	A
Irie-18413	23563313	18413	10	MNCA	A
Irie-18414	23563314	18414	10	MNC	G
Irie-18415	23563315	18415	10	MNC	A
Irie-18416	23563316	18416	10	MNC	T
Irie-18417	23563317	18417	10	MNCA	A
Irie-18423	23563323	18423	10	MNC	A
js4	23563323	18423	10	MNC	A
Irie-18424	23563324	18424	10	MNC	A
js3	23563358	18458	10	finger linker	C
rs61051796	23563395	18495	10	MNCA	A
Irie-18497	23563397	18497	10	MNCA	C
Irie-18498	23563398	18498	10	MNC	A
Irie-18507	23563407	18507	10	MNC	C
Irie-18518	23563418	18518	10	MNC	A
js5	23563442	18542	10	finger linker	C
Irie-18579	23563479	18579	10	MNCA	C
Irie-18635	23563535	18635	10		C

NA = not applicable
ND = not determined
MNC = major nucleotide contact
MNCA = major nucleotide contact adjacent

S = synonymous
NS = non-synonymous
genome and gene positions are 1-based
gene position is based on genome sequence, starting with the "A" of the start codon "ATG" of gene
the population origin for "ND" entries is probably European

allele nt	ref aa	allele aa	type	population	ref homozygote
T	D	D	S	ND	NA
T	R	C	NS	ND	NA
T	G	G	S	ND	NA
T	G	V	NS	Japanese	344
T	S	S	S	Japanese	364
A	R	Q	NS	ND	NA
G	T	S	NS	ND	NA
G	T	S	NS	Japanese	36
G	T	S	NS	European	NA
G	T	S	NS	Yoruban	NA
G	T	R	NS	Japanese	358
A	S	R	NS	ND	NA
G	N	D	NS	ND	NA
T	R	S	NS	ND	NA
T	R	S	NS	European	NA
A	R	S	NS	Japanese	271
A	R	R	S	Japanese	336
A	D	N	NS	Japanese	349
G	H	D	NS	ND	NA
T	H	L	NS	ND	NA
T	R	S	NS	Japanese	256
C	R	S	NS	Japanese	327
T	D	Y	NS	Japanese	330
G	D	G	NS	Japanese	335
G	D	E	NS	Japanese	335
C	K	Q	NS	Japanese	333
G	N	D	NS	Japanese	329
C	N	H	NS	Yoruban, European	NA
T	N	I	NS	Japanese	318
G	E	D	NS	Yoruban	NA
C	S	R	NS	ND	NA
A	S	R	NS	Japanese	339
G	N	D	NS	Japanese	339
A	H	N	NS	Japanese	338
T	R	S	NS	Japanese	303
G	E	D	NS	Yoruban	NA
A	R	S	NS	Japanese	341
T	Y	Y	S	Japanese	342

model CCDS43307.1

heterozygote	allele homozygote	frequency	source	note
NA	NA		dbSNP130	
NA	NA	ND	dbSNP130	
NA	NA	ND	dbSNP130	
1	0	0.0014	Irie et al	sterile only
14	0	0.0185	Irie et al	
NA	NA	ND	dbSNP130	
NA	NA	ND	dbSNP130	
101	239	0.7699	Irie et al	major allele in Japanese
NA	NA	ND	dbSNP130	same as Irie-18097
NA	NA	ND	Ng et al	same as Irie-18097
1	0	0.0014	Irie et al	sterile only
NA	NA	ND	dbSNP130	
NA	NA	ND	dbSNP130	
NA	NA	ND	dbSNP130	
NA	NA	ND	Ng et al	same as rs56001636
85	3	0.1267	Irie et al	
23	0	0.0320	Irie et al	
9	0	0.0126	Irie et al	
NA	NA	ND	dbSNP130	
NA	NA	ND	dbSNP130	
98	3	0.1457	Irie et al	
33	0	0.0458	Irie et al	
30	0	0.0417	Irie et al	
25	0	0.0347	Irie et al	
25	0	0.0347	Irie et al	
27	0	0.0375	Irie et al	
31	0	0.0431	Irie et al	
NA	NA	ND	Ng et al	
42	0	0.0583	Irie et al	
NA	NA	ND	this study	
NA	NA	ND	dbSNP130	
21	0	0.0292	Irie et al	
21	0	0.0292	Irie et al	
22	0	0.0306	Irie et al	
57	0	0.0792	Irie et al	
NA	NA	ND	Ng et al	
10	0	0.0142	Irie et al	
8	0	0.0114	Irie et al	
